# Effects of dietary *Bacillus subtilis* PB6 supplementation to mid-lactation dairy cows on local inflammatory responses during an intramammary lipopolysaccharide challenge

**DOI:** 10.3168/jdsc.2025-0986

**Published:** 2026-02-19

**Authors:** T.H. Swartz, O. Reyes, H.L. Reisinger, A. Celemin Sarmiento, M. Bulnes Larsen, L.K. Mamedova, I.E. Brown-Crowder, J.E. Hergenreder, B.J. Bradford

**Affiliations:** 1 Department of Dairy and Food Science, South Dakota State University, Brookings, SD 57007; 2 Department of Animal Science, Michigan State University, East Lansing, MI 48824; 3 Kemin Industries Inc., Des Moines, IA 50317

## Abstract

The objectives of this study were to evaluate the effects of dietary *Bacillus subtilis* PB6 supplementation on inflammatory mediators in milk during an intramammary LPS challenge in dairy cows. A secondary objective was to determine if repeated LPS challenges induced the development of innate immune memory and immunological tolerance. Mid-lactation Holstein cows (n = 20; 147 ± 48 DIM; 2.2 ± 0.93 parity; 42.1 ± 7.6 kg/d of milk; mean ± SD) were enrolled in a crossover design. Treatments included *Bacillus subtilis* PB6 (BSP) or limestone (control; CON) at 13 g/d, which were top-dressed daily onto a basal lactating TMR for 28-d periods. An intramammary LPS challenge (10 µg, *Escherichia coli* O111:B4) was conducted in a rear quarter on d 24 of each period. Milk samples were collected at 0, 3, 12, and 24 h relative to the intramammary LPS challenge from the challenged quarter to measure SCC and cytokines. For cytokines, milk samples were treated with rennet and centrifuged to obtain whey, and 13 cytokines/chemokines were measured using a multiplex assay (IFNF, IL1A, IL1B, IL4, IL6, IL10, IL17A, IL36RN, CXCL10, CCL2, CCL3, TNF, and VEGFA). Linear mixed models included the fixed effects of treatment, period, and time, and their interactions, and the random effects of cow nested within period, block, and block by period. Dietary BSP supplementation increased SCS at 3 h but decreased SCS at 12 h during period 1 as compared with CON; we found were no treatment effects on SCS during period 2. Dietary BSP increased whey CCL2 (monocyte chemokine) at 3, 12, and 24 h relative to the LPS challenge during period 1; however, during period 2, dietary BSP decreased whey CCL2 at 3 and 12 h as compared with CON. These data suggest that dietary BSP supplementation may have enhanced innate immune training during period 1, and these effects carried over into the CON cows during period 2. Independent of treatment sequence, period 1 had greater concentrations of whey IL1A and IL1B at 3 h following the LPS challenge as compared with period 2, suggesting the development of endotoxin tolerance. In conclusion, our data suggest that dietary BSP supplementation may alter whey CCL2 concentrations, which may have influenced immune cell trafficking to the mammary gland during an intramammary LPS challenge. Period effects point to the development of endotoxin tolerance in cows receiving 2 intramammary LPS challenges.

## Highlights

• We tested dietary *Bacillus subtilis* PB6 effects on LPS-induced mastitis.
• A crossover design was used to evaluate treatment effects on mammary inflammation.
• Dietary BSP increased SCS at 3 hours but decreased SCS at 12 hours following LPS in period 1.
• Of 13 cytokines measured, only MCP-1 (monocyte chemokine) was altered by treatment.
• Dietary BSP had a limited impact on mammary inflammation during an LPS challenge.

## Graphical Abstract

**Figure:**
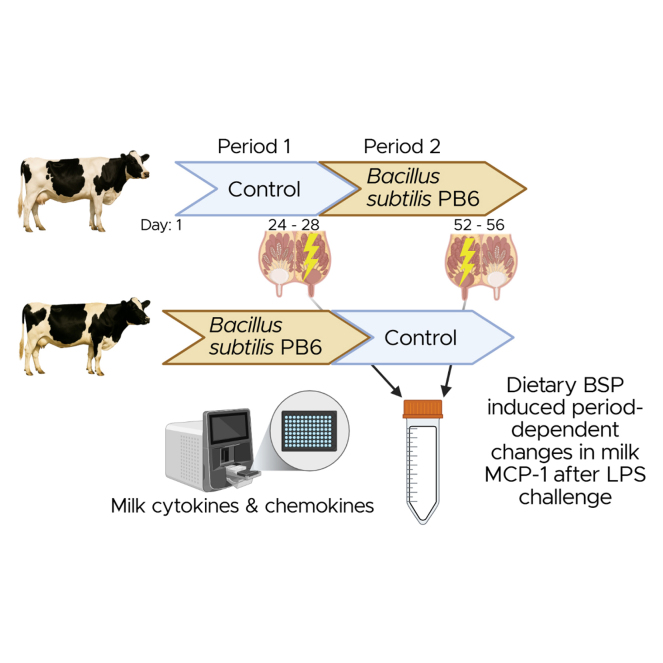
**Summary:** We investigated the effects of dietary *Bacillus subtilis* PB6 (BSP) supplementation on milk inflammatory mediators during an intramammary lipopolysaccharide (LPS) challenge using a crossover design. Dietary BSP supplementation increased somatic cell score (SCS) at 3 hours but decreased SCS at 12 hours following the LPS challenge during period 1, with no effects in period 2. Dietary BSP supplementation increased milk MCP-1 at 3, 12, and 24 hours relative to the LPS challenge during period 1; during period 2, cows receiving dietary BSP supplementation had lower MCP-1 concentrations at 3 and 12 hours compared with the control (CON). Data suggest that dietary BSP supplementation may have enhanced innate immune training during period 1, with carryover effects into CON cows during period 2. Dietary BSP supplementation did not affect milk concentrations of any of the other 12 cytokines measured. Dietary BSP supplementation altered milk MCP-1 concentrations, potentially influencing immune cell trafficking to the mammary gland during an LPS challenge. Created with BioRender.com.

Direct-fed microbials (**DFM**) are feed additives containing live microbes that have been used in the dairy industry as a tool to enhance performance and health. *Bacillus subtilis* PB6 (**BSP**) is a spore-forming DFM that colonizes the small and large intestines and produces antimicrobial compounds for use against pathogens (Lin et al., 2007). Although the effects of DFM supplementation on performance and feed efficiency have been inconsistent (Yoon and Stern, 1995), an area of research where DFM have shown more consistent effects is its ability to improve gastrointestinal (**GI**) health during disease challenges across species. For example, intragastric administration of BSP spores attenuated the local and systemic inflammatory response to colitis in mice (Foligné et al., 2012). In chickens, dietary BSP supplementation improved intestinal health during a *Clostridium perfringens* challenge (Jayaraman et al., 2013). Similarly, in Holstein steers, dietary BSP supplementation reduced pyrexia, serum IL-6, and serum IFN-γ following oral administration of *Salmonella typhimurium* (Broadway et al., 2020). The mechanism(s) behind these responses, though unclear, could be due to BSP supporting beneficial microflora, inhibiting the growth of pathogens in the gut, or a combination of both (Seo et al., 2010).

Another potential mechanism behind BSP responses could be the ability of BSP to directly modulate the immune response (McAllister et al., 2011). As an example, human peripheral blood mononuclear cells cultured with BSP had greater IL10 release while not inducing the production of proinflammatory cytokines such as TNF, indicating an enhanced anti-inflammatory response (Foligné et al., 2012). In addition to directly modulating the immune response, dietary BSP supplementation could influence the development of the immune system in the gut. Trained innate immunity has emerged as a new concept where innate immune cells develop memory after repeated exposures to the same stimuli and enhance defenses during subsequent challenges. Conversely, immunological tolerance is characterized by a dampened inflammatory response following a repeated exposure to the same stimuli (López-Collazo and del Fresno, 2024). The gut microbiome itself, as well as signals derived from the gut microbiome (Maslowski et al., 2009), could be used to train the immune system and tune the inflammatory response (Khan et al., 2016; Negi et al., 2019). Although this phenomenon has been studied more in monogastric animals, the interface between the gut microbiome and the immune system has only just begun to be investigated in ruminants (O'Hara et al., 2020).

Although past studies have assessed the role of BSP on GI health in ruminants (Broadway et al., 2020; Smock et al., 2020), little is known about BSP's ability to modulate immune responses in tissues beyond the GI tract. These studies could inform us of BSP's ability to develop and train the immune system and determine if there are downstream effects on other tissues in addition to the gut, such as the mammary gland in dairy cows. Because mastitis is one of the costliest diseases in the dairy industry (Rasmussen et al., 2024), we used an intramammary LPS challenge to model mastitis caused by a gram-negative pathogen. Therefore, the objectives of this study were to (1) evaluate the effects of dietary BSP supplementation on inflammatory mediators in milk samples during an intramammary LPS challenge, and (2) determine if repeated LPS challenges induced the development of innate immune memory and immunological tolerance in the mammary gland. We hypothesized that dietary BSP supplementation would attenuate inflammation during an intramammary LPS challenge.

This study was conducted at the Michigan State University Dairy Teaching and Research Center (East Lansing, MI), from September 2024 through December 2024. All experimental procedures were approved by the Michigan State University Institutional Animal Care and Use Committee (protocol number PROTO202400242). Mid-lactation Holstein cows (n = 20; 147 ± 48 DIM; parity 2.2 ± 0.93; milk yield 42.1 ± 7.6 kg/d; mean ± SD) were enrolled in a crossover design. Treatments included *Bacillus subtilis* PB6 (BSP; Kemin Industries USA) or limestone (CON) at 13 g/d, which were top-dressed daily onto a basal lactating TMR for 28-d periods. Cows were blocked by parity, DIM, and bovine leukemia virus (**BLV**) status (determined using an ELISA; CentralStar Cooperative). Cows were housed in tiestalls and fed once daily (0800 h). The diet (DM basis) was composed of 41% concentrate mix, 40% corn silage, 14% haylage, and 6% cottonseed (fuzzy). Cows were milked 3 times daily (700, 1500, and 2300 h) in a double-7 herringbone parlor.

An intramammary LPS challenge (10 μg of LPS in 10 mL of PBS; *E. coli* O111:B4, Sigma) was conducted in a rear quarter (left for period 1, and right for period 2) on d 24 of each period. All cows were confirmed to be free of mastitis and had an SCC of <200,000 cells/mL in milk samples collected on d 22 of each period. The infusion was administered 1 h after the morning milking (0900 h). Before infusion, teats were scrubbed with cotton balls soaked in 70% ethanol. The challenge dose (10 mL) was administered in a rear quarter via a teat cannula (Jorgensen Laboratories Inc.). The infusion was dispensed through the teat canal and massaged upward to aid in the dispersion of the LPS into the mammary gland. Afterward, teats were sprayed with a chlorhexidine-gluconate spray (0.4%). Foremilk samples (∼40 mL) were collected just before the LPS challenge (h 0), and then again at 3, 12, and 24 h following the intramammary challenge from the challenged quarter. The first 5 streams of milk were discarded, and foremilk samples were then collected and inverted several times. For SCS, foremilk samples collected after the LPS challenge were diluted 1:10 in PBS and then shipped to a local DHI laboratory (CentralStar Cooperative). The SCC was calculated using the dilution factor and then transformed to SCS as log_2_(SCC/100,000) + 3.

For cytokine analyses, a 2-mL aliquot of the foremilk samples was transferred to a microcentrifuge tube for storage at −80°C before being shipped on dry ice to South Dakota State University (Brookings, SD). First, 1 mL of foremilk was treated with 5 µL of 500 mg/mL rennet (from *Mucor miehei*, Sigma). The foremilk was vortexed, incubated at 37°C for 30 min, vortexed again, and then centrifuged at 2,000 × *g* for 15 min at 22°C. The whey (clear fluid) was collected and then diluted 1:2 in assay buffer to be used for cytokine quantification using Luminex xMAP technology and the Milliplex Bovine Cytokine/Chemokine Panel (Sigma). This panel consisted of 10 cytokines (IFNG, IL1A, IL1B, IL4, IL6, IL10, IL17A, IL36RN, TNF, and VEGFA) and 3 chemokines (CXCL10, CCL2, and CCL3). Quality controls for each cytokine were used on each plate. Samples with cytokine concentrations above the upper limit of detection (**ULOD**) were considered as missing data. Samples with cytokine concentrations below the lower limit of detection (**LLOD**) were imputed by dividing the LLOD by 2 (Helsel, 1990). For each analyte and each plate, the LLOD was defined as the minimal detectable value. To control plate variation, all samples from a specific cow from both periods (treatment and control periods) were assayed together on the same plate.

Data were analyzed using linear mixed models (PROC GLIMMIX, SAS version 9.4, SAS Institute Inc.). The model included the fixed effects of treatment, period, time (repeated measure), and their interactions, with cow nested within period, block, and block by period as random effects. Bovine leukemia virus status (positive or negative) along with its interaction with treatment were tested and retained in the model if *P* ≤ 0.05. Least squares means were separated using the LSMEANS statement with either the SLICE, PDIFF, or SLICEDIFF options in SAS with a Tukey adjustment. The spatial power covariance error structure was used. In all models, residuals were assessed for normality and outliers (PROC UNIVARIATE in SAS). If an outcome was non-normally distributed, logarithmic (base 10) transformation was used. Statistical significance was declared at *P* ≤ 0.05.

For SCS, we observed a treatment by period by time interaction (*P* = 0.01; Figure 1A). When assessing treatment effects within period, dietary BSP supplementation increased SCS at 3 h (*P* = 0.04) but decreased SCS at 12 h (*P* = 0.02) during period 1 as compared with the CON, but had no effect during period 2 (*P* ≥ 0.07). When investigating period effects within treatment groups, CON cows had greater SCS at 12 h (*P* < 0.01) relative to the intramammary LPS challenge during period 1 as compared with period 2. Similarly, BSP cows had greater SCS at 3 h (*P* < 0.01) relative to the intramammary LPS challenge during period 1 as compared with period 2.

**Figure 1:**
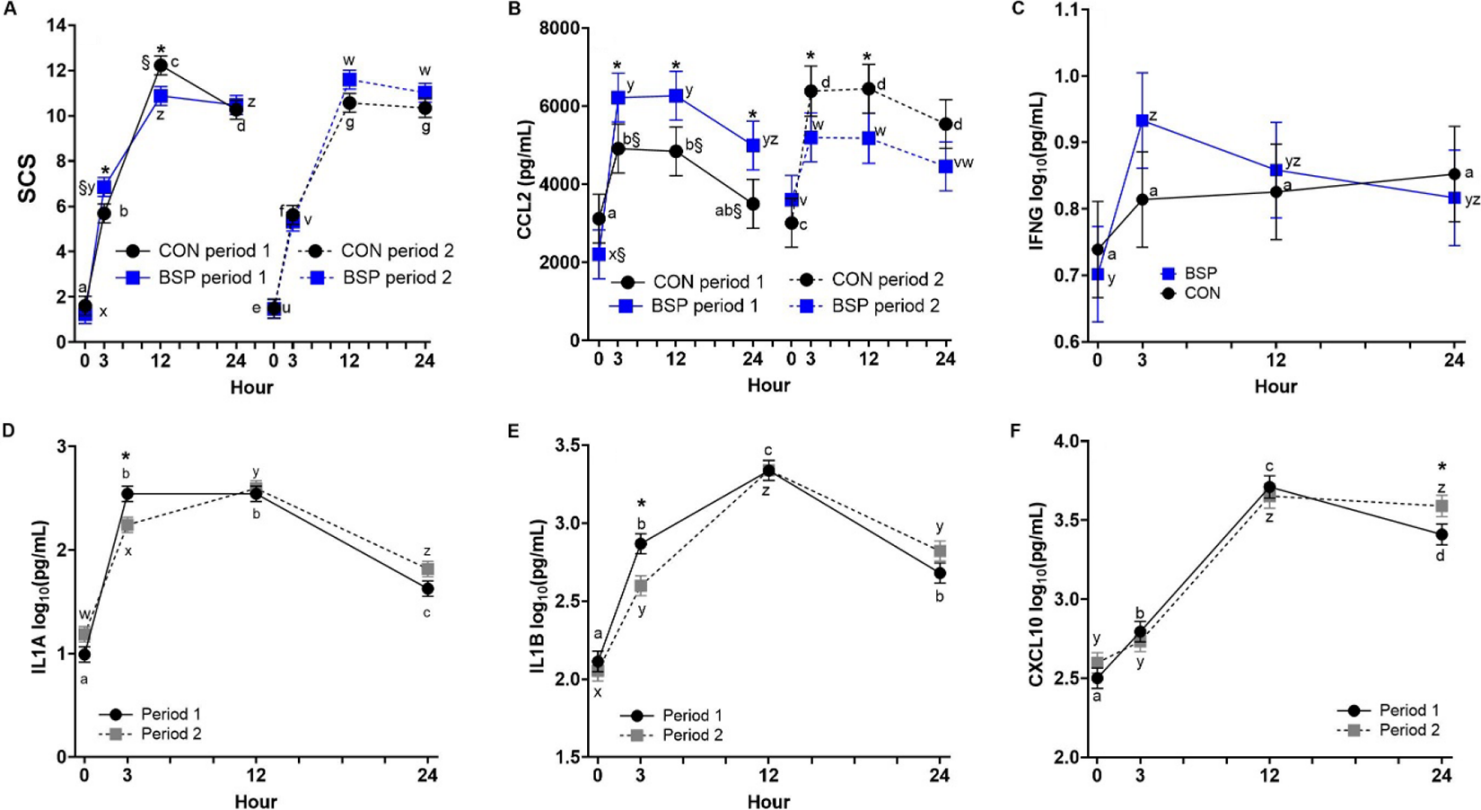
Effects of dietary supplementation of *Bacillus subtilis* PB6 (BSP) to mid-lactation cows on whey cytokine concentrations (pg/mL) measured from milk samples collected at 0, 3, 12, and 24 h relative to an intramammary LPS challenge on d 24 of each period in a crossover design. Treatment by period by time interaction for SCS (A), whey CCL2 (B), treatment by time interaction for IFNG (C), and period by time interactions for whey IL1A (D), IL1B (E), and CXCL10 (F) are illustrated. Different lowercase letters and symbols denote significant effects at *P* ≤ 0.05. For SCS (A): * denotes treatment effects within time and period; a–d denote time effects within period 1 and CON; x–z denote time effects within period 1 and BSP; e–g denote time effects within period 2 and CON; u–w denote time effects within period 2 and BSP; and § denotes period effects within treatment and time. For CCL2 (B): * denotes treatment effects within time and period; a,b denote time effects within period 1 and CON; x–z denote time effects within period 1 and BSP; c,d denote time effects within period 2 and CON; v,w denote time effects within period 2 and BSP; § denotes period effects within treatment and time. For IFNG (C): a denotes time effects within CON and y,z denote time effects within BSP. For IL1A (D), IL1B (E), and CXCL10 (F): * indicates period effects within time; a–d denote time effects within period 1; w–z denote time effects within period 2. Data are presented as LSM ± SE.

For whey CXCL10, 13 samples (8.1%) were above the ULOD (n = 11 at 12 h, n = 2 at 24 h; n = 7 for BSP, n = 6 for CON). For whey CCL2, 2 samples (1.3%) were above the ULOD (n = 1 at 3 h and 12 h; n = 1 for each treatment group). For whey TNF, 7 samples (4.4%) were above the ULOD (n = 6 at 3 h, n = 1 at 12 h; n = 3 for BSP, n = 4 for CON). Conversely, a few samples were below the LLOD, including whey IL1B (n = 1 [0.6%]; n = 1 for BSP) and CCL3 (n = 10 [6.3%]; n = 6 for BSP, n = 4 for CON). The samples below the LLOD were all collected at 0 h relative to the intramammary LPS challenge.

Treatment LSM, SE, and probability levels for whey cytokines are provided in Table 1. We observed a treatment by period by time interaction for whey CCL2 (*P* < 0.01; Figure 1B). When assessing treatment effects within period, BSP cows had greater whey CCL2 concentrations at 3 h (*P* = 0.03), 12 h (*P* = 0.02), and 24 h (*P* = 0.01) relative to the intramammary LPS challenge as compared with CON during period 1. Conversely, during period 2, BSP cows had lower whey CCL2 concentrations at 3 h (*P* = 0.05) and 12 h (*P* = 0.04) relative to the intramammary LPS challenge as compared with CON. When investigating period effects within treatment groups, CON cows had lower whey CCL2 concentrations at 3 h (*P* = 0.02), 12 h (*P* < 0.01), and 24 h (*P* < 0.001) during period 1 as compared with period 2. Moreover, BSP cows had lower whey CCL2 concentrations at 0 h (*P* = 0.02) during period 1 as compared with period 2; no differences were found at the other time points (*P* ≥ 0.07).

**Table 1:** Effects of dietary *Bacillus subtilis* PB6 (BSP) supplementation to mid-lactation cows on whey cytokines (pg/mL) from milk samples collected at 0, 3, 12, and 24 h relative to an intramammary LPS challenge on d 24 of each period in a crossover design^1^

Parameter	Trt LSM		SE	*P*-value						
	CON	BSP		Trt	Period	Trt × period	Time	Trt × time	Period × time	Trt × period × time
IFNG^2^	0.81	0.83	0.062	0.66	0.58	0.86	<0.001	0.04	0.67	0.71
IL1A^2^	1.99	1.93	0.037	0.21	0.97	0.15	<0.001	0.80	<0.01	0.57
IL1B^2^	2.74	2.72	0.033	0.65	0.32	0.03	<0.001	0.73	0.02	0.91
IL4	198	209	19.7	0.56	0.52	0.28	0.21	0.20	0.12	0.73
IL6^2^	3.3	3.3	0.022	0.71	0.18	0.05	<0.001	0.95	0.30	0.37
IL10^2^	2.3	2.4	0.036	0.56	0.07	0.93	<0.001	0.97	0.46	0.14
IL17A^2^	1.01	1.04	0.077	0.56	0.28	0.33	0.02	0.10	0.22	0.30
IL36RN^2^	1.93	1.93	0.041	0.90	0.67	0.90	<0.001	0.67	0.56	0.22
CXCL10^2^	3.1	3.1	0.048	1.00	0.42	0.06	<0.001	0.76	0.04	0.08
CCL2	4,721	4,767	491	0.82	0.02	<0.001	<0.001	0.94	0.75	<0.01
CCL3^2^	2.73	2.74	0.056	0.89	0.97	0.59	<0.001	0.67	0.71	0.20
TNF^2^	4.03	4.13	0.060	0.20	0.65	0.07	<0.001	0.40	0.88	0.33
VEGFA	4,108	3,918	461	0.47	0.14	0.18	<0.01	0.86	0.17	0.81

1 Trt = treatment.

2 Data presented using a logarithmic (base 10) transformation.

The treatment by period interaction was significant for whey IL1B (*P* = 0.03) and IL6 (*P* = 0.05); however, no treatment effects within period were found (all *P* ≥ 0.06). Whey IL1B concentrations were greater in period 1 as compared with period 2 within the BSP group (period 1 vs. period 2 [log_10_], 2.79 vs. 2.64 ± 0.047 log_10_[pg/mL]; *P* = 0.03); however, no period effects were found within the CON group (period 1 vs. period 2 [log_10_], 2.71 vs. 2.77 ± 0.047 log_10_[pg/mL]; *P* = 0.39). Similarly, whey IL6 concentrations were greater in period 1 as compared with period 2 within the BSP group (period 1 vs. period 2 [log_10_], 3.35 vs. 3.25 ± 0.031 log_10_[pg/mL]; *P* = 0.02); however, no period effects were found within the CON group (period 1 vs. period 2 [log_10_], 3.28 vs. 3.30 ± 0.031 log_10_[pg/mL]; *P* = 0.66).

Although the treatment by time interaction was significant for whey IFNG (*P* = 0.04; Figure 1C), no treatment effects were found within each time point (all *P* ≥ 0.08). However, time effects were found within the BSP group, where whey IFNG displayed a spike in concentrations at 3 h (0 h vs. 3 h, *P* < 0.0001) and then declined, becoming similar to baseline values by 12 h (0 h vs. 12 h; *P* = 0.06) and 24 h (0 h vs. 24 h; *P* = 0.32) following the intramammary LPS challenge. This spike and resolution pattern was less apparent in CON, as there were no differences between any time points within the CON group (all *P* ≥ 0.25). For all other whey cytokine concentrations, dietary BSP supplementation did not affect their concentrations (all *P* ≥ 0.20).

We found a period by time interaction (Figure 1D–F) for whey IL1A (*P* < 0.01), IL1B (*P* = 0.02), and CXCL10 (*P* = 0.04). At 3 h following the intramammary LPS challenge, period 1 had greater concentrations of whey IL1A and IL1B as compared with period 2 (both *P* < 0.01). At 24 h following the intramammary LPS challenge, period 1 had lower concentrations of whey CXCL10 as compared with period 2 (*P* = 0.03). Bovine leukemia virus status did not affect any whey cytokine concentrations, except for IL6. Cows that were BLV negative had lower concentrations of whey IL6 as compared with BLV-positive cows (3.24 vs. 3.34 ± 0.022 log_10_[pg/mL], *P* < 0.01). Finally, aside from IL4, all other whey cytokines displayed changes over time (Figure 1, Figure 2).

**Figure 2:**
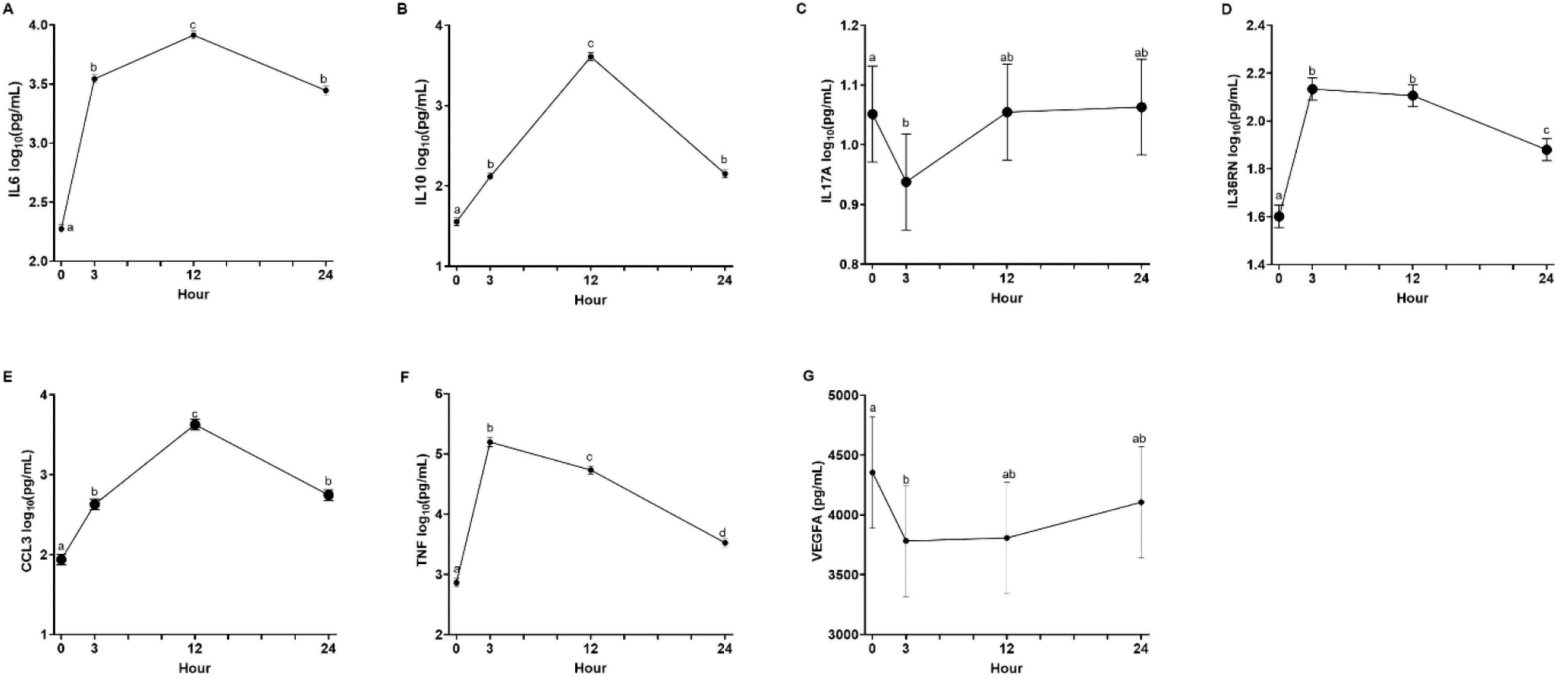
Time effects following an intramammary LPS challenge on whey IL6 (A), IL10 (B), IL17A (C), IL36RN (D), CCL3 (E), TNF (F), and VEGFA (G) cytokine/chemokine concentrations (pg/mL). Milk samples were collected at 0, 3, 12, and 24 h relative to an intramammary LPS challenge. Data are presented as LSM ± SE. Different letters denote significant time effects at *P* ≤ 0.05.

Past studies have found benefits of feeding BSP during GI disease challenges (Broadway et al., 2020; Smock et al., 2020). The primary objective of our study was to determine if feeding BSP affected local inflammatory responses beyond the GI tract by evaluating its effects during an intramammary LPS challenge in dairy cows. Dietary BSP had divergent effects on SCS and did not influence most cytokines measured, suggesting that the health benefits to feeding BSP may be limited in the mammary gland. Dietary BSP supplementation increased SCS at 3 h during period 1, indicating enhanced immune cell trafficking to the mammary gland during the early response to an intramammary LPS challenge, but reduced SCS at 12 h, suggesting attenuation of excessive chemotaxis during peak immune infiltration. Future studies using live mastitis pathogens are needed to clarify the implications of these effects.

Dietary BSP increased CCL2 at h 3, 12, and 24 during period 1. This chemokine attracts immune cells, specifically monocytes, from blood into the site of inflammation, and is produced by a variety of cell types such as monocytes, neutrophils, lymphocytes, and dendritic cells upon stimulation (Singh et al., 2021). Thus, the increase in CCL2 during period 1 could potentially explain the increase in SCS during period 1 at 3 h following the LPS challenge; however, we also found a reduction in SCS at h 12 in period 1 for BSP cows versus CON. The primary immune cells in milk from inflamed mammary glands are neutrophils (Riollet et al., 2002), whereas CCL2 is predominantly a chemoattractant for monocytes. Therefore, potential treatment effects on monocyte recruitment may have been masked by the dominant neutrophil population. Future studies should assess a differential SCC to determine if the increase in CCL2 due to dietary BSP supplementation affected specific immune cell populations in milk.

As mentioned previously, dietary BSP supplementation increased CCL2 during period 1 (h 3, 12, and 24); however, dietary BSP supplementation also decreased CCL2 during period 2 (h 3 and 12). The diverging treatment by period responses for CCL2 are difficult to interpret due to the crossover design of our study. The increase in CCL2 concentrations during period 1 from BSP cows may suggest that BSP supplementation enhanced innate immune training. On the contrary, dietary BSP supplementation reduced CCL2 during period 2, which suggests an alternative mode of action where dietary BSP enhanced the development of endotoxin tolerance. Because of the crossover design of our study, we speculate the more likely explanation for these data is that dietary BSP supplementation may have enhanced innate immune training during period 1, and these effects carried over into CON cows during period 2.

Interferon γ is a cytokine critical for both innate and adaptive immunity to enhance antimicrobial activity from innate immune cells against a variety of pathogens. This cytokine is produced by innate immune cells such as natural killer cells and macrophages as well as by lymphocytes within the adaptive immune system including γδ T cells (both innate and adaptive), T-helper cells, and cytotoxic T cells (Kak et al., 2018). Although we found no differences between BSP and CON at any time point, we did find a treatment by time interaction where time effects within the BSP group were evident, but no time effects were found within the CON group. More specifically, the dietary BSP-supplemented cows had a more robust IFNG response, with a spike occurring early after the LPS challenge (3 h) and resolving by 12 to 24 h later. These time-dependent effects may reflect enhanced immune activation and resolution and support the notion that dietary BSP may have an immunomodulatory role (such as trained innate immunity), although the effect was not large enough to reveal differences between treatment groups.

Previous work has shown that dairy cows primed with an intramammary LPS challenge exhibit reduced inflammation during a subsequent intramammary *E. coli* challenge in the same quarter, indicating the development of endotoxin tolerance (Petzl et al., 2012). In the present study, few period effects were detected, suggesting that the initial intramammary LPS challenge did not substantially alter inflammatory responses during the second challenge. Nevertheless, whey IL1A and IL1B concentrations at 3 h following the intramammary LPS challenges were reduced in period 2 compared with period 1, suggesting a modest and transient endotoxin tolerance effect. Several differences between studies may explain why limited period effects were observed in the present study. Notably, our study employed 2 intramammary LPS challenges, whereas Petzl et al. (2012) used a single LPS challenge followed by an *E. coli* challenge. In addition, Petzl et al. (2012) challenged the same quarter twice, whereas different rear quarters were challenged between periods in the present study, suggesting that endotoxin tolerance may be predominantly locally mediated rather than systemic or influenced by contralateral quarters.

Finally, we provide time course data to characterize the inflammatory response in whey from cows that received an intramammary LPS challenge. Aside from IL4, the other 12 cytokines/chemokines measured were all affected by time, where we found increases (IFNG, IL1A, IL1B, IL6, IL10, IL36RN, CXCL10, CCL2, CCL3, and TNF) or decreases (IL17A and VEGFA) in their concentrations following the LPS challenge. A past study found that an intramammary LPS challenge increased IL1A, IL1B, IL6, IL17A, IL36RN, CXCL10, CCL2, CCL3, TNF, and VEGFA in mammary tissue homogenates following an intramammary LPS challenge, but did not affect IFNG or IL10 (Choudhary et al., 2024). Our results agree with this study for all cytokines except IL10, IL17A, and VEGFA. There are several possible explanations for these differences, including the use of different sample types (i.e., cytokine responses in tissue may differ than those found in milk) or the LPS dose (50 vs. 10 µg in the present study).

A limitation to our study is the potential for biasing our cytokine results due to omitting samples above the ULOD (22 missing datapoints out 2,080 total datapoints) or by imputing the values for samples below the LLOD (11 imputed datapoints out of 2,080 total datapoints), although it should be noted that these samples are distributed across treatment groups fairly evenly. The imputed values for the samples below the LLOD are likely less concerning, as they reflect a lack of immune activation before the intramammary LPS challenge as expected (all samples were collected at 0 h), and these small cytokine concentrations may not be biologically meaningful. Because the assay is multiplexing numerous cytokines, identifying a dilution that can be used to accurately quantify all cytokines is challenging because some cytokines may be over-diluted, whereas other cytokines may not be diluted enough. In future studies of this kind, additional dilution (more than a 1:2 dilution) is recommended for measuring whey CXCL10 and TNF at 3 to 12 h following intramammary LPS challenge.
